# Kidney transplant monitoring by urinary flow cytometry: Biomarker combination of T cells, renal tubular epithelial cells, and podocalyxin-positive cells detects rejection

**DOI:** 10.1038/s41598-020-57524-7

**Published:** 2020-01-21

**Authors:** Nina Goerlich, Hannah Antonia Brand, Valerie Langhans, Sebastian Tesch, Thomas Schachtner, Benjamin Koch, Alexander Paliege, Wolfgang Schneider, Andreas Grützkau, Petra Reinke, Philipp Enghard

**Affiliations:** 1 Charité University Hospital, Berlin, Germany; 2 University Hospital Zurich, Zurich, Switzerland; 3 Goethe University Hospital Frankfurt, Frankfurt, Germany; 4 University Hospital Carl Gustav Carus, Dresden, Germany; 5 German Rheumatism Research Centre, Berlin, Germany

**Keywords:** Diagnostic markers, Renal replacement therapy

## Abstract

Creatinine and proteinuria are used to monitor kidney transplant patients. However, renal biopsies are needed to diagnose renal graft rejection. Here, we assessed whether the quantification of different urinary cells would allow non-invasive detection of rejection. Urinary cell numbers of CD4^+^ and CD8^+^ T cells, monocytes/macrophages, tubular epithelial cells (TEC), and podocalyxin(PDX)-positive cells were determined using flow cytometry and were compared to biopsy results. Urine samples of 63 renal transplant patients were analyzed. Patients with transplant rejection had higher amounts of urinary T cells than controls; however, patients who showed worsening graft function without rejection had similar numbers of T cells. T cells correlated with histological findings (interstitial inflammation p = 0.0005, r = 0.70; tubulitis p = 0.006, r = 0.58). Combining the amount of urinary T cells and TEC, or T cells and PDX^+^ cells, yielded a significant segregation of patients with rejection from patients without rejection (all p < 0.01, area under the curve 0.89–0.91). Urinary cell populations analyzed by flow cytometry have the potential to introduce new monitoring methods for kidney transplant patients. The combination of urinary T cells, TEC, and PDX-positive cells may allow non-invasive detection of transplant rejection.

## Introduction

Although kidney transplantation is the most favorable therapy for end stage renal disease, the risk of rejection remains a constant concern^1^. Allograft rejection leads to a high risk of graft dysfunction, accompanied by a significantly higher probability of chronic failure and graft loss^2–4^. Cellular rejection and humoral rejection have been described to severely impair transplant function and worsening survival prognosis^2^.

Currently, renal transplant function is mainly monitored using creatinine and proteinuria. However, these are only mediocre discriminators for the different renal transplant pathologies. Renal transplant biopsy remains the gold standard for diagnosing transplant rejection, but its use is limited due to its invasive nature. Novel biomarkers hold promise in monitoring different aspects of renal transplant pathology non-invasively, thereby allowing for early detection of transplant rejection and for adjustments in treatment.

In recent years, there has been a tremendous effort to identify novel biomarkers for transplant rejection, including urinary cytokines, binding receptors, proteomics, and genomics^5–7^. However, so far, none of the assessed biomarkers has shown the desired sensitivity and specificity.

Different cells present in the urine may be used as biomarkers, since they likely reflect cellular changes in the transplant and are arguably less variable than upstream inflammatory-signal biomarkers. We have previously reported that urinary T cells analyzed by flow cytometry are an excellent biomarker for intrarenal inflammation^8^. Other groups have already reported on urinary immune cells^9^, including different T cell subsets analyzed with flow cytometry as biomarkers for transplant rejection, with promising results^9–12^. Besides immune cells, the detection of tubular epithelial cells (TEC)^9,10^ and podoctyes^13–15^ have been reported as biomarkers, using urinary sediments in different renal diseases.

Here we hypothesize that cellular signatures of different urinary cells will reflect different elements of the renal transplant pathology. Specifically, assuming that T cells and monocytes/macrophages will reflect intrarenal inflammation; TEC will indicate tubular damage; and podocytes, specifically podocalyxin-positive (PDX-positive) cells, will mirror glomerular pathology, we are interested to know whether the combination of these cells would allow a more precise, non-invasive differentiation of renal transplant rejection from other transplant pathologies, as compared to monitoring only singular cell subsets. In this study, we analyze urinary cell populations of CD4^+^ and CD8^+^ T cells, monocytes/macrophages, TEC, and PDX-positive cells to evaluate correlations with respect to allograft rejection vs. non-rejection. The overall goal of this analysis is to establish a non-invasive diagnostic tool to monitor kidney transplant patients.

## Results

### Urinary tubular epithelial cells and podocalyxin-positive cells can be detected by flow cytometry

**Figure 1:**
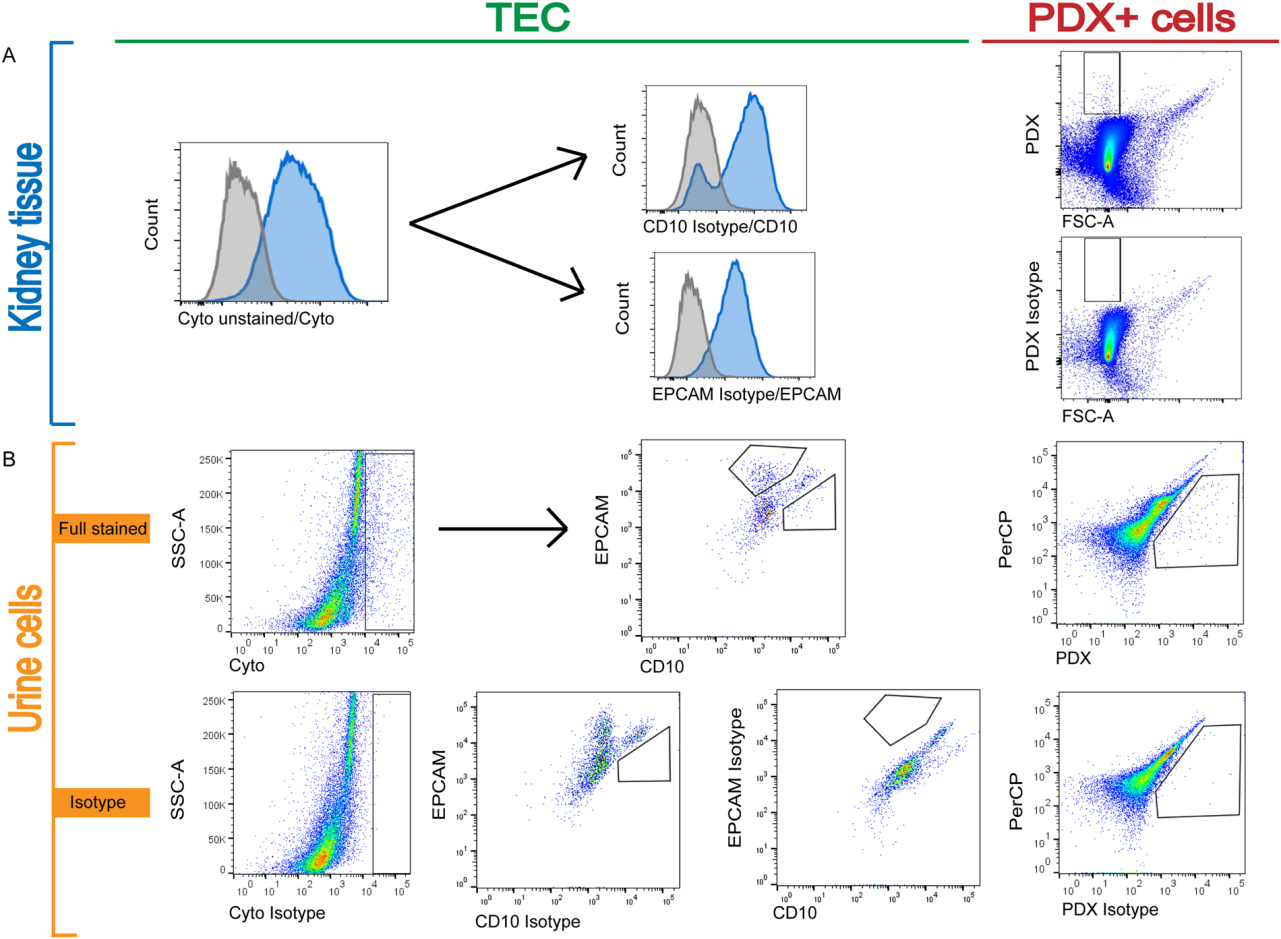
Establishment of a staining assay using human kidney tissue to analyze tubular epithelial cells and podocalyxin-positive cells by flow cytometry. (**A**) Kidney tissue staining. Human kidney tissue from deceased patients was used to establish an applicable antibody panel. TEC biomarker Cytokeratin (intracellular) (grey: unstained, blue: Cytokeratin). Cytokeratin^+^ cells were used to differentiate between proximal (CD10^+^, blue) and distal (EPCAM^+^, blue) TEC; Isotype controls (grey). Podocytes stained with PDX and PDX isotype. (**B**) Urinary isotype controls for TEC and podocytes. Cytokeratin^+^ (intracellular) TEC stained with CD10 and EPCAM; isotype controls for cytokeratin, CD10 and EPCAM. Podocytes stained with PDX and PDX-Isotype. TEC, tubular epithelial cells; PDX, podocalyxin; EPCAM, epithelial cell adhesion molecule.

Urinary TEC were detected using a pan-cytokeratin reactive antibody as lineage marker for epithelial cells, CD10 (also called neutral endopeptidase, NEP, CALLA) as a marker for TEC originating in the proximal tubular system^16,17^ and epithelial cell adhesion molecule (EPCAM) as a marker for distal TEC^18,19^. Therefore, proximal urinary TEC were defined as cytokeratin and CD10 positive cells, and distal TEC as cytokeratin and EPCAM positive cells. Urinary podocalyxin positive cells were analyzed as a surrogate for urinary podocytes. Specificity of the antibody binding was demonstrated using matching isotype controls (Fig. 1).

### Urinary cell composition varies between different graft pathologies

**Figure 2:**
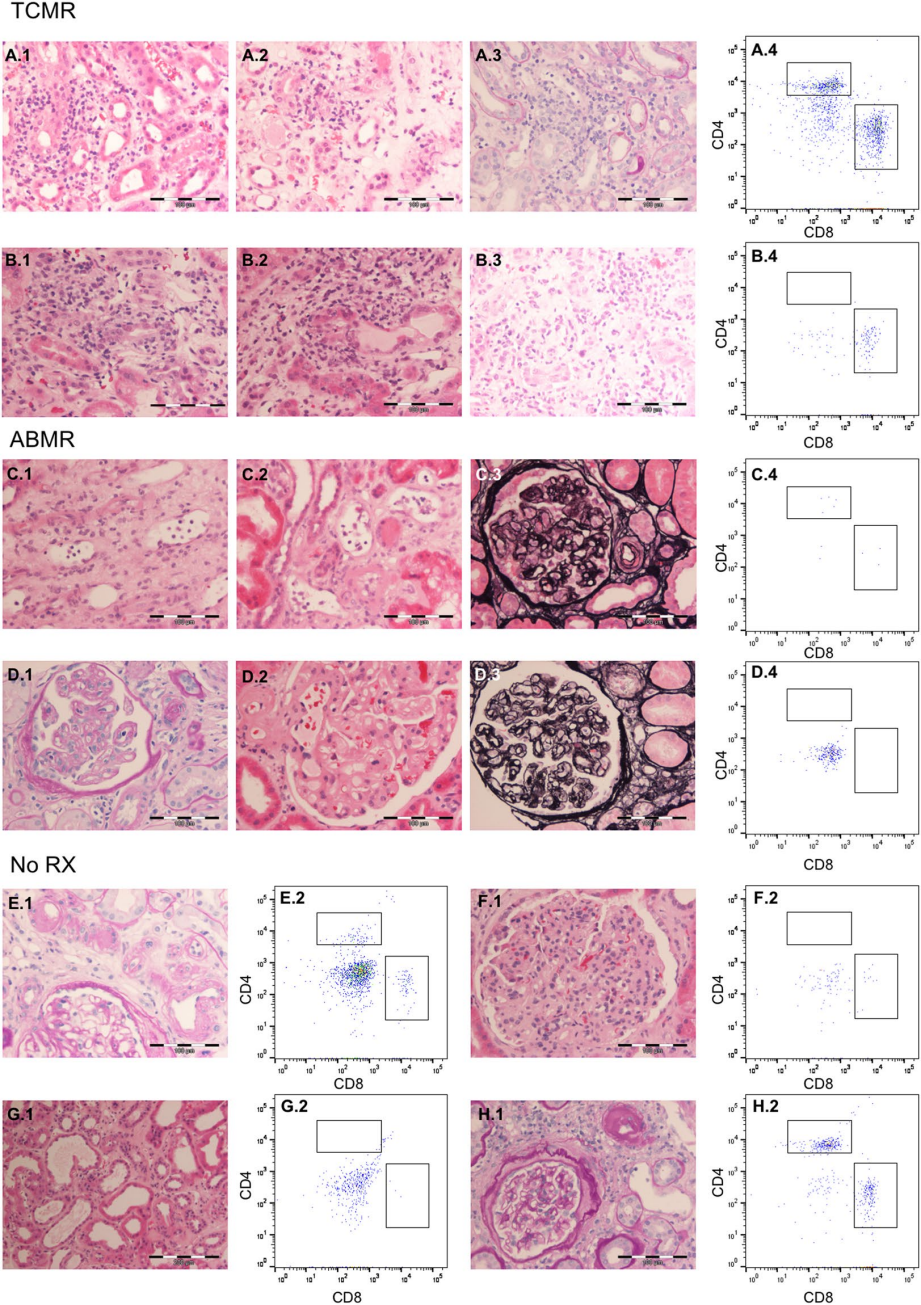
Representative figures of renal biopsies matched with flow cytometry. (**A**) Patient with borderline lesions suspicious for TCMR, **A.1** and **A.2** HE, **A.3** PAS, **A.4**. Flow cytometry analysis of T cells. (**B**) Patient with TCMR, **B.1–B.3** HE, **B.4** Flow cytometry analysis of T cells. (**C**) Patient with ABMR, **C.1** and **C.2** HE, **C.3** Jones’ stain, **C.4** Flow cytometry analysis of T cells. (**D**) Patient with ABMR, **D.1** PAS, **D.2** HE, **D.3** Jones’ stain, **D.4** Flow cytometry analysis of T cells. (**E**–**H**) Patients without rejection, but diverse pathologies. **E.1** and **H.1** PAS, **F.1** and **G.1** HE, **E.2**, **F.2**, **G.2**, **H.2** Flow cytometry analysis of T cells. No RX, no rejection; TCMR, T cell-mediated rejection; ABMR, antibody-mediated rejection; HE, Hematoxylin and eosin stain; PAS, Periodic acid–Schiff reaction.

**Figure 3:**
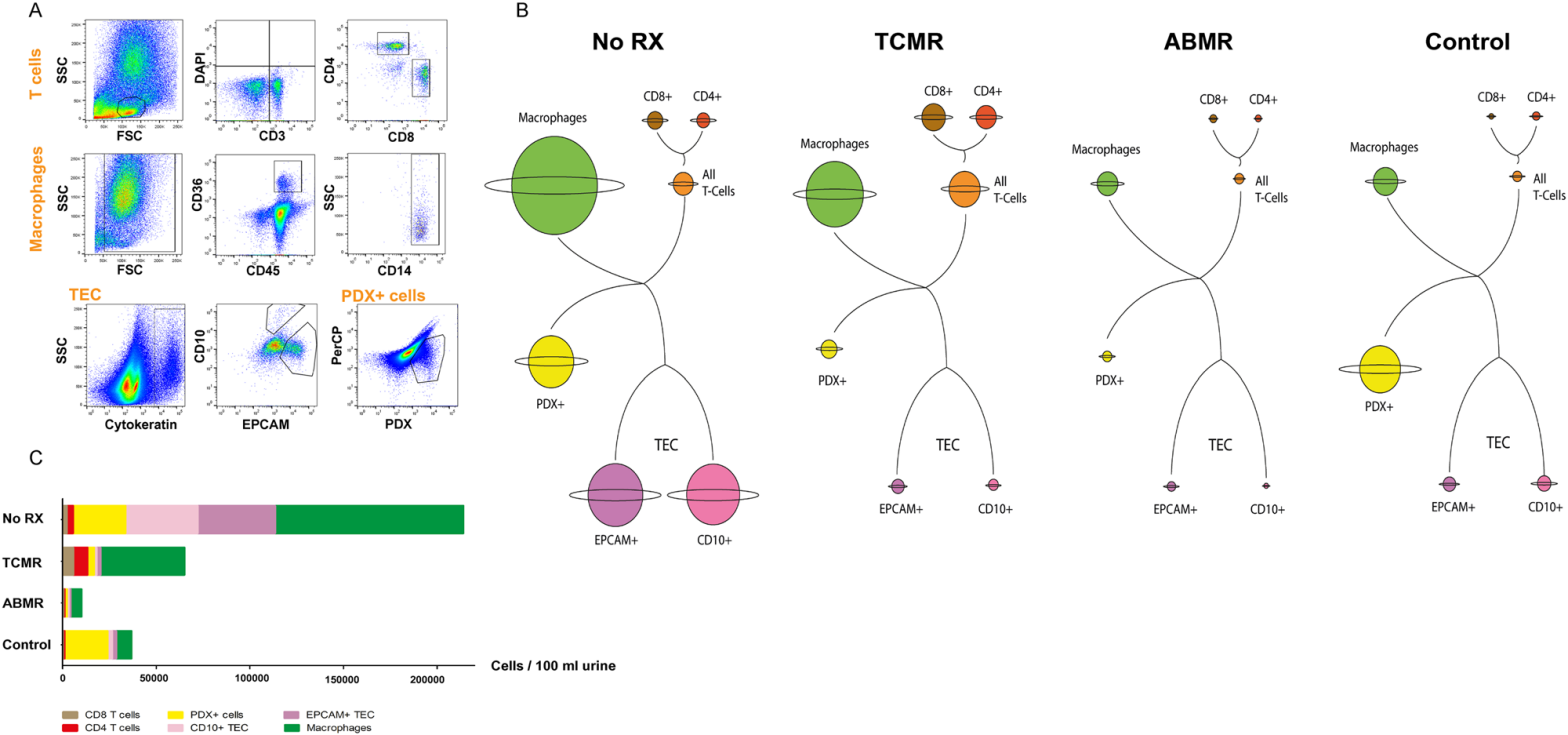
Urinary cell populations in patients with graft deterioration and renal biopsy. (**A**) Urinary T cells, monocytes/macrophages, PDX-positive cells and TEC in a patient with suspected graft rejection analyzed by flow cytometry. The dot plots show a representative gating strategy. (**B**) Patients were allocated to the No RX, TCMR or ABMR group according to the histological diagnosis and a control group of stable kidney transplant patients was analyzed. Planet plots illustrating proportions of urinary cell populations in the analyzed groups. Planet rings are proportional to the standard deviation. (**C**) Subdivision of events (Mean) measured by flow cytometry. SSC – side scatter; FSC – forward scatter; PDX, podocalyxin; EPCAM, epithelial cell adhesion molecule; TEC, tubular epithelial cells; No RX, no rejection; TCMR, T cell-mediated rejection; ABMR, antibody-mediated rejection.

Urine samples of 39 patients with graft deterioration were analyzed by flow cytometry for immune cell populations, PDX-positive cells, and TEC. According to the histological diagnosis of the renal biopsy, 14 patients had T cell-mediated rejection, 7 patients had antibody-mediated rejection, and 18 patients had no signs of rejection (representative biopsy pictures are depicted in Fig. 2). Five patients had inconclusive biopsies and were excluded from the analysis. As shown in Fig. 3, overall urinary cell quantity varied between the described groups. Different cell types were compiled for each group in planet plots: The highest total cell number was found in biopsy patients without rejection (No RX). In these patients, urinary cells consisted mainly of monocytes/macrophages and TEC. The largest T cell population was found in patients with T cell mediated rejection (TCMR). Patients with proven antibody-mediated rejection (ABMR) had the lowest total cell number, as well as the lowest cell numbers in all different cell types.

Urinary TEC, PDX-positive cells, and T cells did not correlate with dipstick results for hemoglobin, protein, or leukocytes.

### Correlation of different urinary cell populations with histopathological lesions

**Figure 4:**
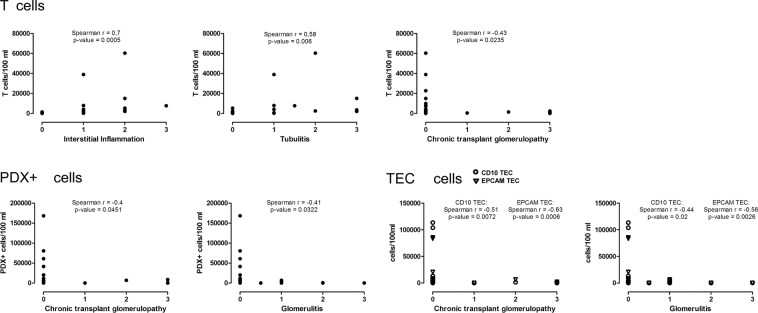
Correlation of urinary cell numbers with histopathological BANFF Classification of 2013. Urinary T cells, TEC and PDX^+^ cells in kidney transplant patients with allograft deterioration. T cell counts correlate with interstitial inflammation and tubulitis. T cell counts negatively correlate with chronic transplant glomerulopathy. TEC and PDX^+^ cells negatively correlate with chronic transplant glomerulopathy and glomerulitis. PDX, podocalyxin; EPCAM, epithelial cell adhesion molecule; TEC, tubular epithelial cells.

To assess our hypothesis that different urinary cell populations reflect specific renal pathologies, we examined the correlations of patients’ urinary numbers with the respective histopathological findings, using the BANFF classification of 2013^20^, which was the most recent at the time of the described analysis (Fig. 4).

As predicted, urinary T cells correlated with levels of interstitial inflammation (p = 0.0005, r = 0.7) and tubulitis (p = 0.006, r = 0.58). Furthermore, T cells negatively correlated with chronic transplant glomerulopathy (p = 0.0235, r = −0.43).

Urinary PDX-positive cells correlated with the histological scores of glomerular injury; however, this correlation was unexpectedly negative. In detail, the quantity of urinary PDX-positive cells negatively correlated with chronic glomerulopathy score (p = 0.0451, r = −0.4) and glomerulitis (p = 0.0322, r = −0.41), and this correlation was mainly driven by high podocyte counts in patients with a score of 0 in the respective BANFF elements.

Urinary TEC negatively correlated with glomerulitis (CD10^+^ TEC: p = 0.02, r = −0.44; EPCAM^+^ TEC: p = 0.0026, r = −0.56) and with chronic transplant glomerulopathy (CD10^+^ TEC: p = 0.0072, r = −0.51; EPCAM^+^ TEC: p = 0.0006, r = −0.63).

Peritubular capillaritis did not correlate with T cells, PDX+ cells or TEC.

### Total amounts of urinary immune cells, tubular epithelial cells, and podocalyxin-positive cells only moderately separate patients with rejection

Next, we assessed whether certain subsets of urinary cells would allow non-invasive detection of acute graft rejection. To this end, we also included a further control group of 24 patients with stable renal graft function without renal biopsy.

**Figure 5:**
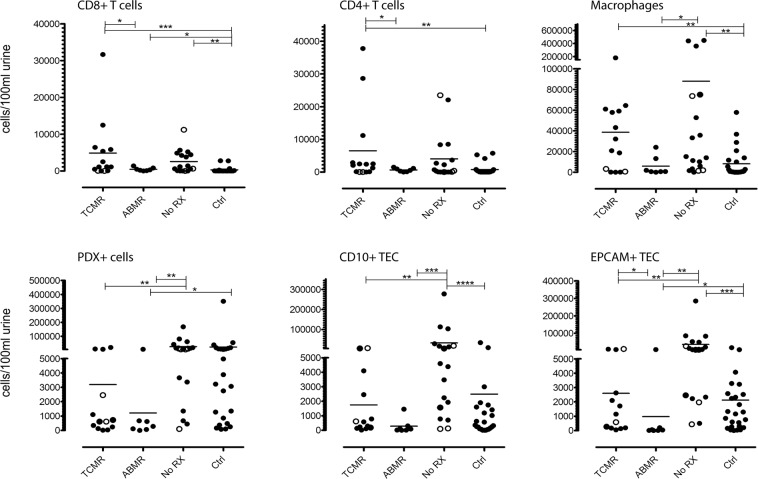
Urinary cell numbers in patients with graft deterioration and renal biopsy. Biopsies of patients with suspected graft rejection were analyzed histologically. Patients were subdivided into three groups according to the diagnosis. A separate group of graft patients with stable renal function not receiving a biopsy is also displayed. Hollow dots symbolize renal biopsies, which were excluded from calculation due to inadequate sample material but did show a suggestive result. *p < 0.05, **p < 0.01 ***p < 0.001 ****p < 0.0001. EPCAM, epithelial cell adhesion molecule; TCMR, T cell-mediated rejection; ABMR, antibody-mediated rejection; No RX, no rejection; Ctrl, control group.

In patients with TCMR, significantly higher amounts of immune cells were observed for CD3^+^CD8^+^ and CD3^+^CD4^+^ T cells (p < 0.05), compared to patients with ABMR. Although the T cells tended to be higher in the TCMR group compared to No RX group, there was no good separation of these groups and no significant difference. Unexpectedly, the patients with No RX had significantly higher urinary counts of PDX-positive cells, proximal TEC, and distal TEC than patients with TCMR (p < 0.01) or ABMR (p < 0.01 for PDX-positive cells, p < 0.001 for CD10^+^ TEC, p < 0.01 for EPCAM^+^ TEC) (Fig. 5).

The control (Ctrl) group consisting of patients with stable renal graft function had significantly lower CD3^+^CD8^+^ (p < 0.001), CD3^+^CD4^+^ (p < 0.01) T cell, and monocytes/macrophage (p < 0.01) counts than TCMR patients (Fig. 5). Compared to the No RX group, patients in the Ctrl group showed lower cell amounts of CD3^+^CD8^+^ T cells (p < 0.01), monocytes/macrophages (p < 0.01), and TEC (CD10^+^ TEC p < 0.0001; EPCAM^+^ TEC: p < 0.001). Like patients with No RX, the Ctrl group showed higher cell counts than the patients with ABMR with respect to the PDX^+^ cells (p < 0.05) and EPCAM^+^ TEC (p < 0.05).

### Increased proportions of T cells per tubular epithelial cells and T cells per podocalyxin-positive cells in patients with allograft rejection

**Figure 6:**
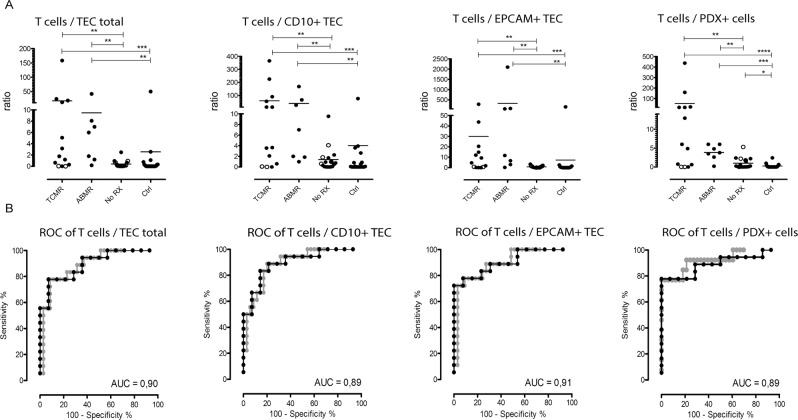
Ratios and ROC curves of urinary cell populations in renal transplant patients with allograft deterioration. (**A**) Patients were allocated to the No RX, TCMR, or ABMR group according to the histological diagnosis. The control group Ctrl includes transplant patients without clinical graft deterioration. Ratios are distinct across patients with TCMR, ABMR, No RX, and the control group. Hollow dots symbolize renal biopsies excluded from calculation due to inadequate sample material but did show a suggestive result. (**B**) ROC curves for biomarker combination of T cells, PDX^+^ cells, CD10^+^ TEC, and EPCAM^+^ TEC. The comparisons are TCMR and ABMR against No RX (black), and TCMR and ABMR against No RX and Ctrl (gray). *p < 0.05, **p < 0.01 ***p < 0.001 ****p < 0.0001. ROC, receiver operating characteristic; AUC, area under the curve; TCMR, T cell mediated rejection; ABMR, antibody-mediated rejection; No RX, no rejection; Ctrl, control group.

As the different patient groups showed different patterns of cells in their urine, we next assessed whether a combination of certain cell subsets would increase the diagnostic yield (Fig. 6). Calculating the ratios of all T cells per TEC, patients with TCMR or ABMR showed a significantly higher ratio compared to the patients with No RX (p < 0.01). No significant difference was observed between patients with TCMR and ABMR.

Similarly, when normalizing the amount of urinary T cells to the amount of PDX-positive cells, a significantly higher ratio was observed in patients with rejection (TCMR or ABMR) than in patients without rejection (p < 0.01). Again, no significant difference was observed between patients with TCMR and ABMR.

Calculating ROC curves, we observed a good segregation of patients with rejection (T cell-mediated or antibody-mediated) from patients who had graft deterioration without rejection (AUC 0.90 for T cells/TEC total, AUC 0.89 for T cells/CD10^+^ TEC, AUC 0.91 for T cell/ECPAM^+^ TEC, AUC 0.89 for T cells/PDX-positive cells).

## Discussion

Kidney allograft rejection is a constant concern in renal transplant patients. Hence, non-invasive biomarkers that can help in monitoring kidney transplant patients and identify rejection are of great interest. Here, we assessed whether the quantification of urinary immune cells, TEC, and PDX-positive cells would allow non-invasive allograft rejection. While the single subsets of urinary cells showed only modest separation of patients with and without rejection, the combination of urinary T cells, TEC, and PDX-positive cells allowed non-invasive detection of patients with acute rejection.

Urinary T cells detected by flow cytometry have been described in various renal diseases and seem to directly reflect renal inflammation, making them a promising biomarker^21–23^. Several groups have assessed urinary T cells as a biomarker for renal transplant rejection and have reported elevated T cells in patients with acute rejection^9–12^. However, urinary T cells alone have not consistently delineated patients with and without rejection in all studies. Therefore, we analyzed whether combining urinary T cells with urinary monocytes/macrophages, TEC, and PDX-positive cells might increase the diagnostic yield of urinary cells in the transplant setting.

Recent research has shown that renal epithelial cells express cytokeratin as stress responders and potential biomarkers^24,25^. In order to distinguish proximal from distal TEC, we used CD10 and EPCAM as they were described by previous authors^16–19^. By combining these markers, we were able to establish detection of urinary proximal and distal TEC.

Urinary podocyte-associated molecules have been reported as a biomarker for various renal diseases^26–28^. Podocalyxin is a surface antigen of podocytes and regulates cell adhesion and morphology^29^. Podocyte damage is supposed to be accompanied by podocyte detachment. Their sheds and fragments are found in the urine as podocalyxin-positive elements^13^. However, podocalyxin is not exclusively expressed by podocytes and therefore is not a specific marker^30^. Furthermore, while staining for TEC and urinary immune cells did allow for identification of circumscribed cell populations (at least in patients with increased amounts), staining for podocalyxin was challenging and did not result in the identification of a clear cell population. Therefore, the cell population referred to as PDX-positive in this paper should not be assumed to be a pure podocyte population and must be interpreted as podocytes with caution.

Assuming that a renal graft rejection induces destruction and detachment of TEC and podocytes, we were surprised to find the largest cell populations in patients without rejection. At least for podocytes, increased shedding after renal transplantation has been reported before, potentially due to podocyte stress^31^. In patients receiving renal biopsy, as expected, the amount of urinary T cells correlated with the histological element, thus reflecting inflammation. However, counter to our prediction, the amount of TEC did not reflect the tubular or glomerular damage observed in the biopsy, whereas PDX-positive cells did negatively correlate with chronic transplant glomerulopathy and glomerulitis. The significance of the high amounts of TEC and PDX-positive cells observed in patients without graft rejection is presently unclear.

In clinical practice, non-invasive detection of graft rejection would enable early diagnosis and would potentially facilitate monitoring and tailoring of the patient’s immunosuppression. Considering that patients without rejection showed high numbers of TEC and PDX-positive cells but low numbers of T cells, we identified a combination of biomarkers as a non-invasive tool for kidney transplant evaluation: the ratio of T cells to TEC or to PDX-positive cells seems capable of distinguishing humoral and acute rejection from no rejection. Specifically, the clearest distinction was achieved by considering the ratio of T cells per EPCAM^+^ TEC (AUC = 0.91). Whether these results can be confirmed in an independent study and can be used to assess treatment responses remains to be determined.

In summary, here we report flow cytometry monitoring of urinary cell populations as a potential method for monitoring kidney transplant patients. Increased amounts of T cells in the urine of patients with rejection support our previous assumption that urinary immune cells mirror intrarenal inflammation. Unexpectedly, urinary TEC and PDX-positive cells did not reflect damage in the respective compartments, and the significance of their presence in urine is presently unknown. Combining counts of urinary T cells, TEC, and PDX-positive cells, however, may provide a useful biosignature for non-invasive detection of renal graft rejection in clinical routine.

## Materials and Methods

### Patients and controls

Urine samples from 63 renal transplant patients were collected and analyzed for urinary cell populations utilizing flow cytometry. Detailed patient characteristics are displayed in Tables 1 and 2, individual immunosuppression details are included as supplemetary information. Of these patients, 39 were admitted due to renal allograft deterioration and underwent renal biopsy as a standard procedure for diagnosing allograft rejection (biopsy cohort). Urine was collected within three days before or after the biopsy.

**Table 1:** Patient Characteristics. Demographic and medical details of kidney transplant patients and rejection therapy. Treatment of rejection started 5 days before and continued for up to 19 days after biopsy. SD, standard deviation; WBC, white blood cell count; RBC, red blood cell count; DoA, Day of Analysis.

Characteristic	T cell-mediated rejection (TCMR)	Antibody-mediated rejection (ABMR)	No rejection in biopsy (No RX)	Control group
Mean age ± SD	49 (±18)	58 (±18)	56 (±13)	47 (±14)
Female/Male	4/10	1/6	6/12	10/14
Living donor/deceased donor transplant	6/6, 2 unknown	2/4, 1 unknown	6/11, 1 unknown	10/14
Primary disease	2x IgA nephropathy, 2x diabetic nephropathy, 1x fanconi syndrome, 2x glomerulonephritis, 1x urinary reflux with megaureter, 6x unknown	1x hypertensive nephropathy, 1x alport syndrome, 1x IgA nephropathy, 1x glomerulonephritis, 1x diabetic nephropathy, 2x unknown	1x glomerulonephritis, 1x lupus nephritis, 4x IgA nephropathy, 1x cardiorenal syndrome, 5x diabetic nephropathy, 1x alport syndrome, 1x hypertensive nephropathy, 2x ADPKD, 2x unknown	4x ADPKD, 2x IgA nephropathy, 2x reflux nephropathy, 1x renal cell carcinoma, 4x glomerulonephritis, 2x alport syndrome, 1x drug-induced nephritis, 2x diabetic nephropathy, 2x hypertensive nephropathy, 1x atypic hemolytic uremic syndrome, 3x unknown
Treatment for rejection	7x methylprednisolone, 3x anti-thymocyte globulin and methylprednisolone, 1x anti-thymocyte globulin, 1x methylprednisolone and IgG therapy, 2x no specific treatment	1x methylprednisolone, 1x cyclophosphamide, 1x plasmapheresis and IgG therapy and anti-thymocyte globulin, 1x methylprednisolone and plasmapheresis, 3x no specific treatment	4x methylprednisolone, 1x anti-thymocyte globulin, 13x no specific treatment	—
Basic immuosuppression	4x tacrolimus + mycophenolate + prednisone, 5x tacrolimus + mycophenolate + methylprednisolone, 3x tacrolimus + mycophenolate, 1x tacrolimus, 1x cyclosporine	3x tacrolimus + mycophenolate, 1x mycophenolate + cyclosporine, 1x tacrolimus + mycophenolate + prednisone, 1x everolimus + mycophenolate + methylprednisolone, 1x tacrolimus + mycophenolate + methylprednisolone	9x tacrolimus + mycophenolate + methylprednisolone, 4x tacrolimus + mycophenolate + prednisone, 2x tacrolimus + mycophenolate, 1x mycophenolate + cyclosporine, 1x tacrolimus + everolimus + methylprednisolone, 1x tacrolimus + azathioprine + methylprednisolone	9x tacrolimus + mycophenolate + methylprednisolone, 9x tacrolimus + mycophenolate, 3x mycophenolate + cyclosporine, 1x everolimus + mycophenolate, 1x mycophenolate + prednisone, 1x none
Level of steriods	2x methylprednisolone 8 mg, 3x methylprednisolone 4 mg, 1x prednisone 125 mg, 1x prednisone 24 mg, 1x prednisone 5 mg, 1x prednisone 4 mg	1x methylprednisolone 4 mg, 1x methylprednisolone 2 mg, 1x prednisone 2 mg	9x methylprednisolone 4 mg, 1x methylprednisone 8 mg, 1x methylprednisolone 24 mg, 1x prednisone 2.5 mg, 1x prednisone 4 mg, 1x prednisone 7.5 mg, 1x prednisone 250 mg	1x methylprednisolone 10 mg, 1x methylprednisolone 8 mg, 4x methylprednisolone 4 mg, 3x methylprednisolone 2 mg, 1x prednisone 5 mg
CNI Level median (range)	7.90 (9.74) ng/ml	5.6 (0.7) ng/ml	13.97 (21.6) ng/ml	6.19 (11.86) ng/ml
GFR median (range) DoA	23.57 (77) ml/min/1.73 m²	21.28 (32) ml/min/1.73 m²	23.61 (54) ml/min/1.73 m²	53.34 (93) ml/min/1.73 m²
Creatinine median (range) DoA	7.82 (44.95) mg/dl	3.1 (2.39) mg/dl	3.66 (10.14) mg/dl	1.54 (3.39) mg/dl
Urea median (range)	95.37 (208.8) mg/dl	133.71 (96) mg/dl	105.33 (165) mg/dl	57 (119) mg/dl
Urinary protein-creatinine ratio median (range) DoA	547.48 (1807.6) mg/g creatinine	1357.2 (1918) mg/g creatinine	1523.44 (4588) mg/g creatinine	426.42 (4298) mg/g creatinine
Urine volume ± SD	111 (±39) ml	120 (±50) ml	84 (±45) ml	76 (±34) ml
Proteinuria median (range)	30 (100) mg/dl	65 (300) mg/dl	22.5 (2000) mg/dl	0 (100) mg/dl
Leucocyturia median (range)	0 (125) WBC/μl	0 (0) WBC/μl	0 (70) WBC/μl	0 (70) WBC/μl
Erythrocyturia median (range)	200 (200) RBC/μl	5 (200) RBC/μl	80 (200) RBC/μl	0 (80) RBC/μl
Days after transplant median (range)	66.5 (9524)	3368 (1833)	105 (5970)	2067 (7349)

**Table 2:** Patient characteristics: Histopathological lesions in transplant biopsies. Median and standard deviation of BANFF scores, fibrosis and tubular atrophy. TCMR, T cell-mediated rejection; ABMR, antibody-mediated rejection; No RX, no rejection.

Biopsy result	TCMR	ABMR	No RX
Interstitial inflammation (i)	2.0 (0.6)	0.5 (0.7)	0.0 (0.9)
Tubulitis (t)	1.3 (0.8)	0.0 (0.0)	0.0 (0.0)
Glomerulitis (g)	0.0 (0.8)	1.0 (0.5)	0.0 (0.3)
Peritubular capillaritis (ptc)	0.0 (0.9)	0.5 (0.9)	0.0 (0.0)
Chronic glomerulopathy (cg)	0.0 (1.1)	3.0 (0.7)	0.0 (0.3)
Intimal arteritis (i)	0.0 (0.8)	0.0 (0.7)	0.0 (0.6)
Arteriolar hyalinosis (ah)	0.0 (1.0)	3.0 (0.4)	1.5 (1.4)
Mesangial expansion (mm)	0.0 (0.9)	0.5 (0.5)	0.0 (0.8)
Fibrosis	1.3 (0.9)	1.0 (0.0.)	0.4 (0.6)
Tubular atrophy	1.6 (1.0)	1.3 (0.5)	0.8 (0.5)

The current study divided these patients into three groups according to their histological diagnosis: patients who had been classified as affected by acute cellular rejection were considered the TCMR group; patients with proven acute or chronic humoral rejection constituted the ABMR group; and samples without rejection were classified as the No RX group.

Patients with uncertain histological diagnosis due to insufficient biopsy material were excluded from the analysis; however, they are displayed here as separate data points within the diagnostic categories suggested by their respective renal pathologists. Meanwhile, a handful of particular diagnoses excluded biopsy patients from this study: membranous glomerulonephritis, urinary tract infection, allergic interstitial nephritis, focal segmental glomerulosclerosis, and recurrent IgA nephropathy.

An additional cohort of 24 transplant patients with stable graft function was analyzed as a control group. Stable graft function was defined by stable serum creatinine (fluctuations less than 25%) or creatinine decrease on regular outpatient clinic visits, and no admittance related to issues regarding kidney transplant function during the 6 months prior to urine analysis. Of these, 6 cases deserve special mention. One control patient showed an elevation in creatinine 1 month prior to analysis, which resolved within 1 week; the creatinine elevation was of unclear significance. Furthermore, in 5 patients with long-stable transplant function (median years after transplant = 8.39), creatinine had not been measured 6 months prior to urine analysis because outpatient visits were scheduled less frequently; nonetheless, these 5 patients met the criteria of stable creatinine and absence of hospital admittance. All 6 of these cases remained in the Ctrl group.

Urine collection started in March 2016 and ended in March 2017. Samples were collected from patients of the Department of Nephrology, Charité University Hospital, Berlin. Informed consent was obtained from all patients for study participation. Furthermore, Patients agreed and gave written consent to participate and to the publication of the findings in a scientific journal. Moreover, our method of biomarker staining was tested on kidney tissue harvested postmortem from deceased patients with the consent of their relatives for postmortem donation of a small kidney sample for research purpose. The Charité University review board granted ethical approval (Charité EA1/152/16 and EA 2/045/18), and the study was conducted according to the ethical guidelines both at our institution and of the Helsinki Declaration.

### Establishment of a method for detecting tubular epithelial cells and podocalyxin-positive cells via flow cytometry by staining kidneys from deceased patients

To establish a staining protocol for the urinary detection of proximal TEC, distal TEC, and PDX-positive cells, human kidneys donated by deceased individuals were digested within 24 hours after death and used. Tissue was digested in RPMI medium containing collagenase VIII and DNase for 30–60 minutes at 37 °C. Red blood cell lysis was performed by BD Pharm Lyse (BD, Franklin Lakes, NJ, USA). Following this, cells were stained with viability dye and separated into dead and living cells. Staining protocol and flow cytometry analysis were equal, as described below.

### Staining and flow cytometry

Within 8 hours after collection, a urine dipstick test for each sample was performed (Only nitrite-negative samples were used for analysis.), and urine samples were centrifuged (280 × *g*, 4 °C, 8 minutes). The pellet was resuspended in phosphate-buffered saline (PBS)/bovine serum albumin (BSA). Prior to the staining, peripheral blood monoclonal cells were isolated using Ficoll-Paque (Density 1.073 g/mL, Sigma-Aldrich, Hamburg, Germany). Fixation with 2% paraformaldehyde was performed for the staining of TEC and podocytes. The following monoclonal antibodies and conjugates were used for staining: CD3-PerCP (Clone: BW264/56, Isotype mouse IgG2), CD4-PE-Vio770 (Clone: VIT4, Isotype mouse IgG2a), CD8-APC-Vio770 (Clone: 135/80, Isotype mouse IgG2), CD45RO-VioGreen (Clone: 5B1, Isotype mouse IgG2), CD36-PE (AC106, Isotype mouse IgG2a), CD14-FITC (Clone: TÜK4, Isotype: mouse IgG2), Cytokeratin-FITC (Clone: CK3-6H5, Isotype: mouse IgG1), CD10-PE-Vio770 (Clone: 97C5, Isotype: Mouse IgG1), EPCAM-APC-Vio770 (Clone: HEA-125, Isotype: mouse IgG1), Podocalyxin-PE (Clone: REA246) (all Miltenyi Biotec GmbH, Bergisch Gladbach, Germany), Alexa Fluor 647 anti-cytokeratin-FITC (Clone: C-11, Isotype: Mouse IgG1) (BioLegend, San Diego, USA).

Cells were incubated with antibody cocktails for 15 minutes at 4 °C in the dark. PBS/BSA containing 10% human IgG (Flebogamma; Grifols, Langen, Germany) was utilized to block unspecific binding. In addition, Saponin (S7900 Sigma-Aldrich, Hamburg, Germany) was used for intracellular staining of cytokeratin (0.25 g in 50 ml PBS). Immediately before flow cytometric analysis, 4′,6-Diamidine-2′-phenylindole dihydrochloride (DAPI) (Sigma-Aldrich, Hamburg, Germany) was added to exclude dead cells (only for unfixed samples). Cell numbers were calculated and normalized to a volume of 100 mL urine. Samples were analyzed using a MACS Quant Analyzer (Miltenyi Biotec GmbH, Bergisch Gladbach, Germany). Datasets were analyzed using FlowJo 10.3 (Tree Star, Ashland, Oregon, USA).

### Statistical analysis

In consideration of this trial as an exploratory study, the Mann-Whitney test was used to calculate differences in urinary cell populations. For all tests, the significance threshold is set at p < 0.05. All medians, means, Mann-Whitney tests, receiver operating characteristic (ROC) curves, and Spearman correlations were calculated using GraphPad Prism 5 (GraphPad Software, San Diego, California, USA). The formula for the creation of planet plots was root (Mean/3.14)*2 for the planet’s size and root ((Mean + standard deviation))/3.14)*2 for the size of the ring (the ellipse, representing the standard deviation). Adobe Illustrator CC (Adobe Systems Incorporated, San Jose, California, USA) was used for presentation of planet plots.

## Supplementary information

Supplementary Dataset 1.
